# Spatiotemporal wind speed forecasting using conditional local convolution and multidimensional meteorology features

**DOI:** 10.1038/s41598-024-78303-8

**Published:** 2024-10-31

**Authors:** Meng Wang, Juanle Wang, Mingming Yu, Fei Yang

**Affiliations:** 1 State Key Laboratory of Resources and Environmental Information System, Institute of Geographic Sciences and Natural Resources Research of Chinese Academy of Sciences, Beijing, 100101 China; 2 College of Resources and Environment, University of Chinese Academy of Sciences, Beijing, 100049 China; 3 Jiangsu Center for Collaborative Innovation in Geographical Information Resource Development and Application, Nanjing, 210023 China; 4 School of Computer Science and Engineering, Beihang University, Beijing, 100191 China

**Keywords:** Wind resources, Spatiotemporal wind forecasting, Local convolution kernel, Recurrent neural network, Inner Mongolia region, Climate sciences, Atmospheric science, Atmospheric dynamics

## Abstract

Wind speed prediction is crucial for precisely wind power forecasting and reduced maintenance costs. Highland regions, which possess a considerable wind potential, present complex meteorological conditions, making wind speed prediction challenging. Traditional weather forecasting relies on complex statistical methods and extensive prior knowledge. While recent deep learning models have improved prediction accuracy, they often assume uniform influence weight structure, limiting model effectiveness. This study introduces an enhanced Conditional Local Convolution Recurrent Network (CLCRN) model to improve spatiotemporal wind speed forecasting using multidimensional meteorological inputs such as temperature, pressure, and dew point, alongside wind components. This model addresses uniform influence model weight issue by redesigning convolution kernels to better capture local meteorological features and integrating multiple influencing factors. Our model consistently achieves lower Mean Absolute Error (MAE) and Root Mean Squared Error (RMSE) values across various prediction intervals (3, 6, 9, and 12 h) compared to other models, supported by the meteorological station data from 2019 to 2021. Furthermore, the spatial distribution of the local convolution weights aligns with local wind velocity patterns in Inner Mongolia, enhancing model interpretability. These results demonstrate potential for practical applications in renewable energy planning and wind dynamics simulation.

**Supplementary Information:**

The online version contains supplementary material available at 10.1038/s41598-024-78303-8.

## Introduction

Near-surface wind has significant potential as an environmentally friendly and renewable energy source, garnering extensive international recognition^1^. Variations in surface wind velocity caused by climate change, primarily manifested as global warming, are crucial for the effective utilisation and risk mitigation of wind energy resources and the security of ecosystems^2,3^. Wind speed variations can affect wind turbine efficacy and wind energy generation^4,5^. Wind energy resources exhibit an inherently intermittent nature over brief time intervals, presenting considerable obstacles for grid planners and operators^6^. Accurate wind speed prediction can promote optimise the placement and operation of wind turbines, improve weather-dependent logistical operations, and contribute to the sustainable development of renewable energy sources. Spatiotemporal wind speed forecasting is an urgent scientific and technological challenge for global and local, especially with special and different geographical feature areas.

The unique topography of the Mongolian Plateau, creates complex and sustained wind flows ideal for wind energy generation. High-altitude areas experience stronger winds, making parts of the plateau promising for wind energy potential^7^. Wind speed prediction relies on three main model categories: physical, statistical, and artificial intelligence (AI) models^8,9^. Physical models simulate atmospheric variables for forecasting but are not ideal for short-term predictions owing to high computational demands^10^. Statistical approaches optimise parameters using historical data and have shown promising results for short-term forecasting^11^. However, these methods depend on extensive domain knowledge for effective feature extraction, which limits their generalisation. With advancements in computer science, deep learning models like RNNs^12^, CNNs^13^, and GNNs^14^gain prevalence due to their superior performance and ability to automatically extract features^15^, overcoming the limitations of high computational demands and extensive domain knowledge required by physical and statistical methods^16,17^.

However, there are still significant challenges in deep learning based meteorological forecasting models. Irregular sampling of meteorological signals hampers the effectiveness of classical convolutional neural networks (CNNs), which are designed for regular mesh grid signals in Euclidean domains, such as 2-D planar images. Signals are typically collected from sensors that are unevenly distributed across non-planar manifolds; for example, meteorological sensors are scattered across land and ocean without structured mesh grids, resulting in meteorological data being represented as spherical rather than planar signals. Meteorological stations that measure wind speed are often unevenly distributed, and the data they produce are typically spherical signals, which are not suitable for traditional CNNS^18^. Additionally, meteorological patterns vary significantly across different geographic locations, encompassing extreme weather events, making it challenging to develop a unified modelling approach. Various methods have been proposed for processing spherical signals, including multiview projection^19^and graph-based spherical convolution^20^. In recent years, methods combining Graph Neural Networks (GNN) and Recurrent Neural Networks (RNN) have achieved significant success in wind speed prediction^21–23^. These methods effectively address dynamic graph issues in spatiotemporal prediction by abstracting graph structures in static spatial slices and accumulating embeddings over time, such as spatio-temporal graph convolutional network (STGCN)^24^, diffusion convolutional recurrent neural network (DCRNN)^25^, and adaptive graph convolutional recurrent network (AGCRN)^26^. However, due to the unique characteristics of meteorological forecasting, GNN architecture is not ideally suited for wind speed prediction tasks. Existing methods for graph convolutional spatio-temporal wind speed prediction are often based on models originally developed for traditional traffic flow prediction tasks, making them less effective at handling the irregular spatial distribution and high spatio-temporal dependency characteristics of meteorological data. Current graph convolutional approaches for spatiotemporal wind speed prediction typically employ fixed convolutional kernels, which struggle to effectively capture diverse meteorological patterns associated with different geographical locations. In GNNs, the weights between adjacent nodes can vary significantly based on connection direction and distance. But in meteorology, each local point is influenced by factors from all directions, and the influence weights among points within a local geographic region should be similar, rather than varying by direction and distance.

To better simulate local meteorological feature consistency, several studies have redesigned model convolution kernels to meet region-specific meteorological and geographic features, ensuring three key requirements: similar local features, shared convolution kernels for adjacent local features, and geographical differences. These adaptations have achieved state-of-the-art results. Lin et al^18^. developed a conditional local kernel (CLC) structure that uses local coordinate representations and center node locations as inputs to capture the smoothness and location-specific features of local patterns. This structure is specifically designed for spatiotemporal meteorological forecasting tasks. This core approach leverages the assumption of smoothness in local spatial patterns by approximating the convolution kernel with a feedforward network, considering the distance and orientation weights to adapt to the irregular spatial distribution of meteorological data. Then Lin et al. proposed CLCRN model integrates CLC structure into an RNN architecture^18^, effectively capturing local meteorological patterns and temporal dynamics, offering state-of-the-art performance. However, current models tend to predict each weather parameter—such as temperature, humidity, and surface wind components—independently, evaluating their accuracy separately rather than integrating all factors into a comprehensive analysis. For instance, temperature predictions often rely solely on historical temperature data without incorporating other relevant variables like humidity. This segmented approach limits model ability to capture the complex interactions among different meteorological factors. Therefore, there is a need for models that integrate related parameters holistically to enhance prediction accuracy and provide a more complete understanding of weather dynamics.

In order to address this limitation, this study proposes advanced CLCRN model to enhance the model’s applicability in meteorological and geographic domains. We integrate five characteristics closely related to wind components to jointly complete three-hour interval wind forecasting tasks, including air temperature, dew point temperature, air pressure relative to mean sea level, and surface wind components (wind direction and wind speed). In order to empirically validate the findings, this study employ a diverse range of historical weather data from 25 meteorological stations situated in northern Inner Mongolia, China. The CLCRN model’s local convolution kernel is employed to visualize the interaction weights between adjacent stations, revealing a significant northwest-southeast directional axis in weight distribution. The model is tested with projections ranging from immediate (3-hour) to extended (12-hour) forecasts, thereby demonstrating its capability for both short-term and long-term predictions. The enhanced model demonstrates superior performance in terms of lower mean absolute error (MAE) and root mean squared error (RMSE) at 3-, 6-, 9-, and 12-hour intervals compared to other models with 5-dimensional inputs. The enhanced deterministic wind speed prediction algorithm proposed in this study will facilitate more effective utilization of wind energy, enhance wind speed forecasting services for the Mongolian Plateau region.

## Materials and methods

### Dataset and problem statement

**Fig. 1:**
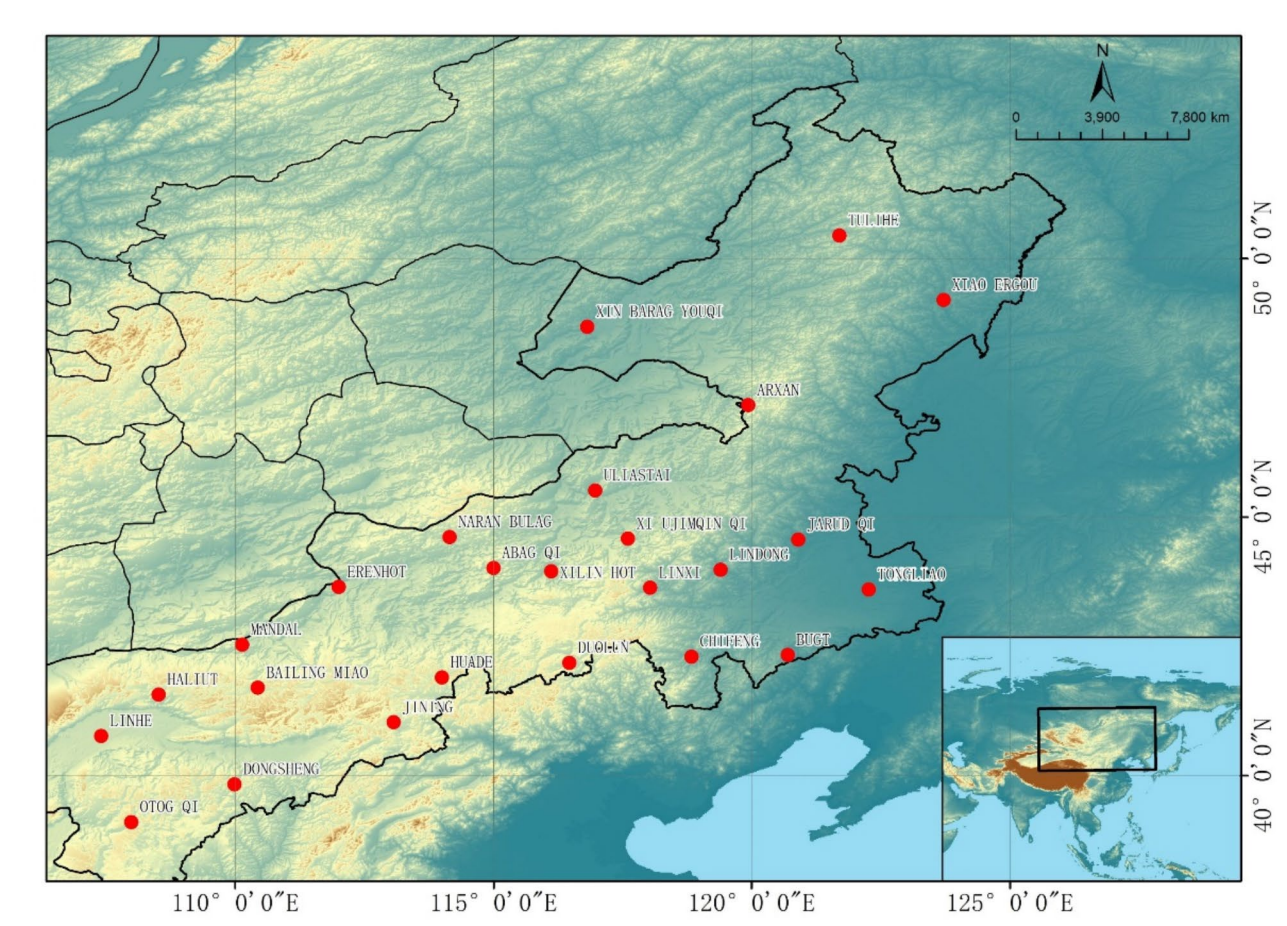
Twenty-five measurement stations in Inner Mongolia used to construct the dataset. The figure was created using ArcGIS 10.7 (https://www.esri.com/en-us/arcgis/about-arcgis/overview)^27^.

The data for this study is sourced from twenty-five meteorological stations in the southeastern area of the Mongolian Plateau (shown in Fig. 1). The data spans from January 1, 2019, to December 31, 2021, and includes five critical meteorological parameters: wind velocity, wind direction, air temperature, dew point temperature, and atmospheric pressure, recorded every three hours. These parameters are directly related to wind dynamics:

- Air Temperature: It indicates the current temperature of the air, which can affect the wind speed and direction due to thermal gradients.
- Dew Point Temperature: It provides a measure of atmospheric moisture, influencing air density and pressure differences.
- Air Pressure: It offers insights into pressure gradients, which are the fundamental drivers of wind.
- Surface Wind Components: It includes both the wind direction and speed, essential for direct modelling and forecasting. We integrate wind speed and direction by decomposing the wind speed records into their respective components using the sine and cosine of the wind direction angles. The wind direction, combined with the wind speed parameter, is decomposed into its sine ‘u’ component (eastward component of the wind speed) and cosine ‘v’ component (northward component of the wind speed) to fully capture the circular characteristics, thereby enhancing the overall understanding of wind dynamics in our model.

All meteorological data are downloaded from the National Climatic Data Center (NCDC) website (http://www.ncdc.noaa.gov/), a database that provides free access to NCDC’s archive of station history information, global historical weather and climate data.

The primary objective of this study is to predict wind speed in the southeastern area of the Mongolian Plateau using historical meteorological data. Specifically, we used multi-factor meteorological data from the past 12 h to forecast wind speeds for the subsequent 12-hour period. To construct the dataset, we implemented a sliding window approach with a window size of 4 and a stride of 1, resulting in a total of 8,699 samples. Each time record is spaced 3 h apart, with each sample consisting of four consecutive time steps, thereby effectively capturing the requisite temporal dynamics. We slid the window from the beginning of the dataset to the last available time record, moving one step at a time to construct the sample data. This strategy is applied uniformly across all features, including wind speed, temperature, humidity, and surface pressure, ensuring that each variable accurately reflects the dynamic relationships within the historical data. The dataset was then divided into three subsets: 70% for training, 10% for validation, and 20% for testing. The validation set was specifically used for model selection, while the test set was designated for evaluating the model’s forecasting performance. Afterward, Z-score normalization was applied to the multi-factor inputs, standardizing the data by subtracting the mean and dividing by the standard deviation for each feature, resulting in a mean of 0 and a standard deviation of 1. This ensured consistency and comparability across features.

In this paper, we present the formalization of wind forecasting as a spatio-temporal task. Let $\:\mathcal{G}=\left(\varvec{V},\varvec{E},\varvec{A}\right)$ represent the signals on the sphere manifold, where $\:\varvec{V}$ is the set of N nodes, $\:\varvec{E}$ is the set of edges, and $\:\varvec{A}$ is the adjacency matrix. The multi-factor signals at time $\:t$ of the nodes on $\:\mathcal{G}$ are denoted by $\:{\varvec{X}}_{t}\in\:{R}^{N\times\:d}$, where $\:d$ is the dimension of the input signals. Given historical meteorological data over $\:P$ time steps $\:\mathcal{X}=\{{\varvec{X}}_{{t}_{1}},{\varvec{X}}_{{t}_{2}},\dots\:,{\varvec{X}}_{{t}_{P}}\}\in\:{R}^{N\times\:d\times\:P}$, our goal is to predict the states of the wind speed components of N nodes in the u and v directions over the next consecutive $\:Q$ time steps, denoted as $\:\mathcal{Y}=\{{\varvec{Y}}_{{t}_{P+1}},{\varvec{Y}}_{{t}_{P+2}},\dots\:,{\varvec{Y}}_{{t}_{P+Q}}\}$. The problem is defined as follows:

1 $$\:\{{\varvec{X}}_{{t}_{1}},\dots\:,{\varvec{X}}_{{t}_{P}};\mathcal{G}\}\:\overrightarrow{\mathcal{F}\left(\cdot\:\right)}\:\:\:\{{\varvec{Y}}_{{t}_{P+1}},\dots\:,{\varvec{Y}}_{{t}_{P+Q}};\mathcal{G}\},$$

where $\:\mathcal{F}$ is the mapping function from $\:\varvec{X}$ to $\:\varvec{Y}$ we aim to learn.

### Model architecture

#### Overview

**Fig. 2:**
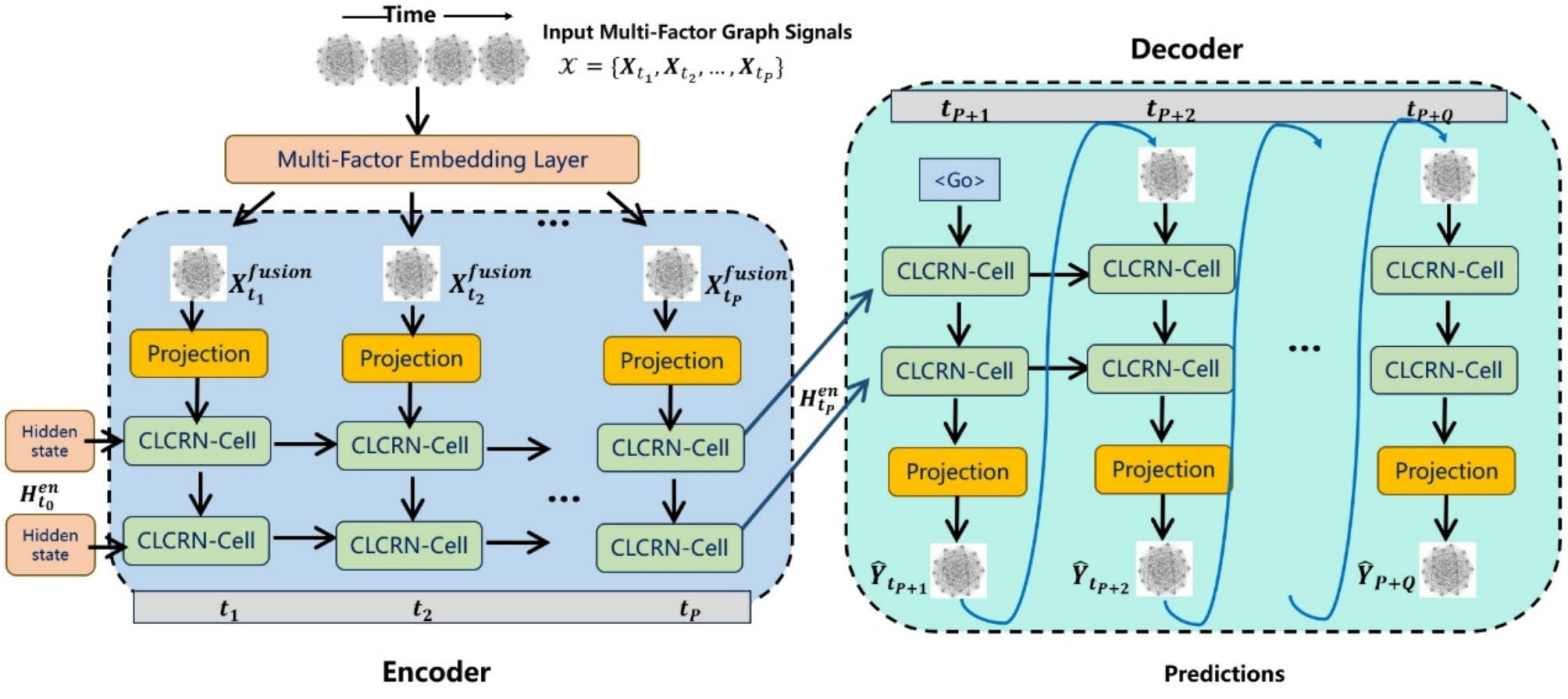
Overall workflow and architecture of our enhanced CLCRN model. The input signals are processed through the Multi-factor Embedding Module to obtain fused features of various geophysical factors. These fused features are then modelled by the encoder to capture the spatiotemporal characteristics of all historical observations. Finally, the embedded features are sent to the decoder, which outputs future predictions using an autoregressive approach.

As shown in Fig. 2, the model consists of three main components: a multi-factor embedding module that integrates inputs such as wind speed and dew point pressure, an encoder that performs spatiotemporal modelling on the embedded features, and a decoder that generates predictions using an autoregressive approach based on the spatiotemporal features from the encoder^18^. The encoder-decoder structure is composed of two layers of CLCRN cells. In this section, we first introduce the multi-factor embedding module, then the CLCRN-Cell, and finally the entire encoder-decoder framework.

#### Multi-factor embedding module

Unlike previous methods that rely solely on past wind speed values without considering broader meteorological factors, this study integrates multiple factors to enhance wind speed prediction. We incorporate five key characteristics for a comprehensive three-hour forecasting task: air temperature, dew point temperature, air pressure relative to mean sea level, and surface wind components (wind direction and speed).

**Fig. 3:**
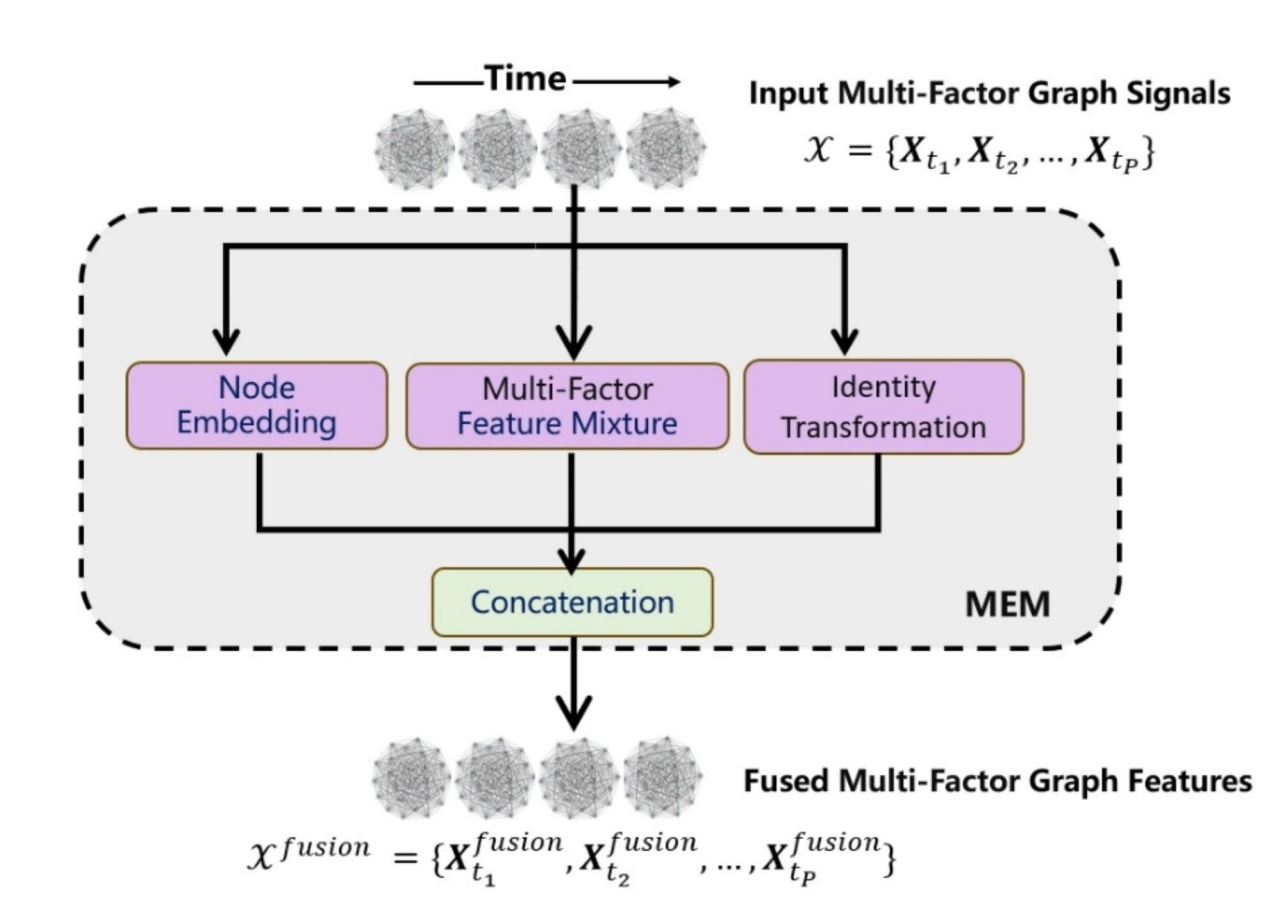
Overall architecture of Multi-factor Embedding Module.

These factors are embedded within a Multi-factor Embedding Module(MEM), serving as node features in a spatiotemporal graph. As illustrated in Fig. 3, the layer processes input signals over time through three parallel pathways: node embedding, multi-factor feature mixture, and identity transformation.

1. Node Embedding Pathway: It uses a set of learnable parameters $\:{\varvec{X}}_{t}^{node}$ to capture the spatial characteristics of each node, ensuring that spatial dependencies are effectively represented.
2. Multi-factor Feature Mixture Pathway: It uses a linear layer to blend input channels, including wind speed components (u and v), atmospheric pressure, and dew points, into an embedding space, producing the mixed feature $\:{\varvec{X}}_{t}^{mix}$. This captures interactions among different factors, enabling the model to exploit complex relationships within the data.
3. Identity Transformation Pathway: It preserves the original input features $\:{\varvec{X}}_{t}$, maintaining the integrity of the initial data.

The outputs from these three pathways are concatenated to form a fused feature representation. For a single time step input $\:{\varvec{X}}_{t}$, the output after processing through these three pathways is given by:

4 $$\:{\varvec{X}}_{t}^{fusion}=Concat\left({\varvec{X}}_{t}^{node},{\varvec{X}}_{t}^{mix},{\varvec{X}}_{t}\right)\in\:{R}^{N\times\:{d}^{{\prime\:}}}$$

*where N is the number of node and* $\:{d}^{{\prime\:}}=21$ *is the feature dimension.*

Thus, given historical meteorological data over P time steps, $\:\mathcal{X}=\{{\varvec{X}}_{{t}_{1}},{\varvec{X}}_{{t}_{2}},\dots\:,{\varvec{X}}_{{t}_{P}}\}\in\:{R}^{N\times\:d\times\:P}$, and the model outputs a sequence of fused features:

5 $$\:{\mathcal{X}}^{fusion}\:=\{{\varvec{X}}_{{t}_{1}}^{fusion},{\varvec{X}}_{{t}_{2}}^{fusion},\dots\:,{\varvec{X}}_{{t}_{P}}^{fusion}\}\in\:{R}^{N\times\:{d}^{{\prime\:}}\times\:P}$$

#### CLCRN-cell architecture

**(1) Conditional Local Convolution kernel**.

We employ Conditional Local Convolution (CLC) to handle graph message aggregation on the spherical manifold of the Earth in order to capture local spatial dependencies and patterns.

##### Establish local space

For efficient convolution operations, the neighbors of each node on the sphere are projected into a local Euclidean space using isometric mappings. Formally, for a node *i*, the neighbors *j* is mapped as.

6 $$\:{\varvec{x}}_{j}^{{i}^{{\prime\:}}}=\text{localMap}\left({\varvec{x}}_{i},{\varvec{x}}_{j}\right),$$

**Kernel Compution**: the kernel is coumputed by angle and distance scaling factor, and the MLP output:

7 $$\:\mathcal{T}\left({\varvec{x}}_{j}^{{i}^{{\prime\:}}};{\varvec{x}}_{i}\right)=\underset{\text{The angle scaling factor}}{\underbrace{\frac{{{\uppsi\:}}_{j}^{{i}^{{\prime\:}}}}{2{\uppi\:}}}}\cdot\:\underset{\text{The distance scaling factor}}{\underbrace{\text{exp}\left(-\frac{{\left({{\uprho\:}}_{j}^{{i}^{{\prime\:}}}\right)}^{2}}{{\uptau\:}}\right)}}\cdot\:\underset{\text{MLP output}}{\underbrace{\text{MLP}\left(\left[{\varvec{x}}_{j}^{{i}^{{\prime\:}}},{\varvec{x}}_{i}\right]\right)}}$$

This MLP takes into account the relative coordinates $\:{\varvec{x}}_{j}^{{i}^{{\prime\:}}}$of the projected neighbor nodes and the central node $\:{\varvec{x}}_{i}$, adaptively learning the specific feature patterns of each local region. In our model, the $\:\tau\:$is a learned parameter during training, and we use a 3-layer MLP with neuron counts of^6,8,10^.

##### Message passing

The aggregated message for a node 𝑖 is computed by summing the weighted features of its neighbors.

8 $$\:\left({\Omega\:}\star\:{\nu\:}_{\left(i\right)}F\right)\left({\varvec{x}}_{i}^{E}\right)={\sum\:}_{{\varvec{x}}_{j}\in\:\nu\:\left(i\right)}\mathcal{T}\left({\varvec{x}}_{j}^{{i}^{{\prime\:}}},{\varvec{x}}_{i}\right)F\left({\varvec{x}}_{j}\right),$$

Where the $\:v\left(i\right)$ is the neighborhood set of center $\:i$. F is a function mapping each point on sphere to its feature vector.

**(2) CLCRN-Cell architecture**.

The CLCRN-Cell has a similar structure to the GRU-Cell. We replace the fully connected layers in GRU-Cell with the Conditional Local Convolutions, resulting in the CLCRN-Cell. Specifically, for node $\:i$, the computation through the CLCRN-Cell is defined as follows:

9 $$\:{r}_{i,t\:}={\upsigma\:}\left({\Omega\:}\star\:{\upnu\:}\left(i\right)\left[{\varvec{F}}_{i,t},{\varvec{H}}_{i,t-1}\right]{\varvec{W}}_{r}+{b}_{r}\right)$$

10 $$\:{u}_{i,t\:}={\upsigma\:}\left({\Omega\:}\star\:{\upnu\:}\left(i\right)\left[{\varvec{F}}_{i,t},{\varvec{H}}_{i,t-1}\right]{\varvec{W}}_{u}+{b}_{u}\right)$$

11 $$\:{\varvec{C}}_{i,t}={tan}h\left(\varOmega\:\star\:\nu\:\left(i\right)\left[{\varvec{F}}_{i,t}\:,\:\left({r}_{i,t\:}\odot\:{\varvec{H}}_{i,t-1}\right)\right]{\varvec{W}}_{C}+{b}_{C}\right)$$

12 $$\:{\varvec{H}}_{i,\text{t}}={u}_{i,t-1}\odot\:{\varvec{H}}_{i,t-1}+\left(I-{u}_{i,t}\right)\odot\:{\varvec{C}}_{i,t}$$

Where $\:{\varvec{F}}_{i,t}$ and $\:{\varvec{H}}_{i,\text{t}}$ denote the input and output of node $\:i$ at time $\:t$, while $\:{r}_{i,t\:}$and $\:{r}_{i,t\:}$ are reset gate and update gate of node $\:i$ at time $\:t$. $\:{\varvec{W}}_{r}$, $\:{\varvec{W}}_{u}$ and $\:{\varvec{W}}_{C}\:\:$are weights, and $\:{b}_{r}$, $\:{b}_{u}$, $\:{b}_{c}$ are bias. Similar to the GRU-Cell, we utilize the CLCRN-Cell to construct the recurrent neural network layers in both the Encoder and Decoder. In our model, the number of layers is set to 2, and the hidden units in the CLCRN-Cell is 32.

#### Encoder-decoder framework

The encoder-decoder framework in our model is designed to effectively capture the spatiotemporal dependencies in the meteorological data, enabling accurate wind speed predictions. This section details the structure and function of the encoder and decoder components.

The encoder consists of multiple layers of Conditional Local Convolution Recurrent Network (CLCRN) cells, which process the input multi-factor graph signals to capture the temporal and spatial dynamics of the data. Initially, a projection layer is applied to the input signals at each time step, transforming them into a suitable representation:

13 $$\:{\varvec{F}}_{t}=Projection\left({\varvec{X}}_{t}^{fusion}\right)$$

Once the projection is completed, the encoder updates its hidden state $\:{\varvec{H}}_{t}^{\text{e}\text{n}}$ for each time step $\:t$. This update utilizes both the input graph features $\:{F}_{t}$and the previous hidden state $\:{\varvec{H}}_{t-1}^{\text{e}\text{n}}$.

14 $$\:{\varvec{H}}_{t}^{\text{e}\text{n}}=2\text{CLCRN-Cell}\left({\varvec{H}}_{t-1}^{\text{e}\text{n}},{\varvec{F}}_{t},\mathcal{G}\right)$$

As the sequence progresses, the encoder processes the series of input representations $\:\{{\varvec{F}}_{{t}_{1}},\dots\:,{\varvec{F}}_{{t}_{P}}\}$. This sequential processing ultimately yields the final hidden state $\:{\varvec{H}}_{{t}_{P}}^{\text{e}\text{n}}$, encapsulating the spatiotemporal information from the entire input sequence. The overall encoding process is thus formulated as:

15 $$\:{\varvec{H}}_{{t}_{P}}^{\text{e}\text{n}}=\text{Encoder}\left({\varvec{F}}_{{t}_{1}},\dots\:,{\varvec{F}}_{{t}_{P}},{\varvec{H}}_{{t}_{0}}^{en}\right)$$

The decoder utilizes the final hidden state from the encoder to generate future predictions. i.e., $\:{\varvec{H}}_{{t}_{P}}^{\text{d}\text{e}}={\varvec{H}}_{{t}_{P}}^{\text{en}}$. Similar to the encoder, the decoder is composed of CLCRN cells and operates in an autoregressive manner. Starting with the encoder’s final hidden state $\:{\varvec{H}}_{{t}_{P}}^{\text{en}}$, the decoder predicts the future graph features:

16 $$\:{\varvec{H}}_{t+1}^{\text{d}\text{e}}=2\text{CLCRN-Cell}\left({\varvec{H}}_{t}^{\text{d}\text{e}},{\widehat{\varvec{Y}}}_{t},\mathcal{G}\right)$$

17 $$\:{\widehat{\varvec{Y}}}_{t+1}=\text{Projector}\left({\varvec{H}}_{t+1}^{\text{d}\text{e}}\right)$$

Thus, the sequence of predicted future winds can be expressed as:

18 $$\:\{{\widehat{\varvec{Y}}}_{{t}_{P+1}},\dots\:,{\widehat{\varvec{Y}}}_{{t}_{P+Q}}\}=\text{Decoder}\left({\varvec{H}}_{{t}_{P}}^{\text{e}\text{n}},\mathcal{G}\right)$$

## Results

### Evaluation metrics

To rigorously assess the quality of the predictive outputs generated by the proposed model, we employed a comprehensive array of statistical metrics. These metrics were selected because of their capacity to collectively evaluate various aspects of forecast accuracy and reliability.

MAE offers insight into the average magnitude of errors in the predictions, irrespective of their direction. It is defined as:

19 $$\:MAE=\frac{1}{Q}\sum\:_{i=1}^{Q}\left|{{\varvec{Y}}_{{t}_{P+i}}-\:\widehat{\varvec{Y}}}_{{t}_{P+i}}\right|,$$

where $\:{\varvec{Y}}_{{t}_{P+i}}$ represents the actual value, $\:{\:\widehat{\varvec{Y}}}_{{t}_{P+i}}\:$is the predicted value.

RMSE provides a measure of the average-squared discrepancies between the predicted and observed values, thereby penalising larger errors more severely. It is defined as:

20 $$\:RMSE=\sqrt{\frac{1}{Q}\sum\:_{\:i=\:1}^{Q}{\left({{\varvec{Y}}_{{t}_{P+i}}-\:\widehat{\varvec{Y}}}_{{t}_{P+i}}\right)}^{2}}.$$

These two metrics combined form a robust framework for evaluating a predictive model, enabling a thorough understanding of its performance characteristics. By examining these metrics in unison, we can discern not only the accuracy of the model’s predictions but also the consistency of its performance across the range of data it has been tasked with forecasting.

To ensure a fair comparison of performance, we adopt consistent experimental settings for both RNN-based models (TGCN, DCRNN, GConvGRU, CLCRN) and attention-based models (STGCN, MSTGCN, CLCSTN), as shown in Table 1. For RNN models, GRU is used as the temporal modelling module, while different spatial modelling methods are applied. The Hidden Unitsand Block Number are set to 32 and 2, respectively. For attention-based models, Conv-1D serves as the temporal modelling module, with spatial methods including Vanilla GCN, ChebConv, and CLC. The Hidden Units and Block Number are set to 64 and 4, respectively.

**Table 1:** Parameters and model structures for different models. Spatial and temporal refer to the different methods used for spatial and temporal modelling, respectively. Hidden units and Block Number indicate the number of hidden units and the number of blocks in the spatial-temporal module.

Model	Spatial	Temporal	Hidden-units	Block-num
TGCN	Vanilla GCN	GRU	32	2
DCRNN	DiffConv	GRU	32	2
GConvGRU	ChebConv	GRU	32	2
CLCRN	CLC	GRU	32	2
STGCN	Vanilla GCN	Conv-1D	64	4
MSTGCN	ChebConv	Conv-1D	64	4
CLCSTN	CLC	Conv-1D	64	4

### Benchmarking against alternative models

For empirical validation, we utilised an extensive historical dataset collected from 25 stations to forecast wind speeds across multiple temporal horizons. The performance metrics included the RMSE and MAE, providing a comprehensive assessment of the predictive accuracy and reliability of the model. MAE offers an average of the absolute discrepancies between the predicted values and actual observations, whereas RMSE provides a squared average, emphasising larger deviations.

Table 2 presents the MAE and RMSE values for various models at 3-, 6-, 9-, and 12-hour prediction intervals using the five meteorological parameters. The numerical values in the table reflect the mean forecast errors of the models, with lower scores indicating more precise predictions. The results show that our enhanced CLCRN model consistently achieves lower MAE and RMSE values across all prediction intervals compared with the other models. Specifically, our enhanced model demonstrates significant improvements in both short-term (3 h, 6 h) and long-term (9 h, 12 h) predictions, indicating its robustness and accuracy in forecasting wind dynamics. This underscores the sophisticated ability of the model to capture complex patterns and dependencies in meteorological data, thereby providing more accurate and reliable forecasts.

**Table 2:** MAE and MSE values for various models(DCRNN, Diffusion Convolutional recurrent neural network; GCoNVGRU, Graph Convolutional Gated recurrent unit; TGCN, temporal graph Convolutional Network; STGCN, Spatio-Temporal Graph Convolutional Network; MSTGCN, multi-step temporal graph Convolutional Network; CLCSTN, conditional local convolution spatio-temporal network; and CLCRN, Conditional Local Convolution Recurrent Network) at 3-, 6-, 9-, and 12-h prediction intervals using 5 weather characters.

Model	MAE				RMSE			
	1 step (3 h)	2 steps (6 h)	3 steps (9 h)	4 steps (12 h)	1 step (3 h)	2 steps (6 h)	3 steps (9 h)	4 steps (12 h)
DCRNN	1.15	1.26	1.34	1.39	1.59	1.74	1.84	1.91
GCoNVGRU	1.15	1.26	1.34	1.39	1.59	1.74	1.84	1.90
TGCN	1.53	1.55	1.57	1.59	2.06	2.09	2.12	2.14
STGCN	1.82	1.82	1.82	1.82	2.40	2.40	2.40	2.40
MSTGCN	1.42	1.46	1.49	1.50	1.92	1.98	2.02	2.04
CLCSTN	1.10	1.18	1.23	1.27	1.53	1.63	1.70	1.76
CLCRN	**1.09**	**1.17**	**1.22**	**1.26**	**1.51**	**1.61**	**1.68**	**1.74**

Then, this study also compares the performance of the CLC-based model using all five meteorological parameters (5D input) against the performance when using only the sine ‘u’ and cosine ‘v’ components of wind speed (2D input), as shown in Table 3. This table presents the MAE and RMSE values for the model at 3-, 6-, 9-, and 12-hour prediction intervals, highlighting the differences in forecast accuracy when utilizing a full set of meteorological data versus a reduced set focused solely on wind speed components. In Table 3, the results using 2D inputs indicate that the CLCRN model predictions are slightly less accurate than those of the CLCSTN model. Although the difference in performance is minimal, it suggests that the CLCRN model relies more heavily on multidimensional meteorological data to achieve improved prediction accuracy. This dependence on a broader range of inputs underscores the importance of integrating multiple meteorological parameters to enhance model performance. The results reinforce that utilizing comprehensive datasets can provide better understanding of weather dynamics, ultimately leading to better wind speed forecasting accuracy.

**Table 3:** MAE and MSE values for conditional local kernel-based models (CLCRN and CLCSTN) at 3-, 6-, 9-, and 12-h prediction intervals using only 2 wind characters (sine ‘u’ and cosine ‘v’ wind speed components).

Model	Metrics	1 step (3 h)	2 steps (6 h)	3 steps (9 h)	4 steps (12 h)
CLCRN	MAE	1.17	1.24	1.30	1.34
	RMSE	1.60	1.70	1.78	1.83
CLCSTN	MAE	1.13	1.21	1.28	1.32
	RMSE	1.56	1.68	1.76	1.82

Among the benchmarking results presented in Tables 2 and 3, Our enhanced model with five-dimensional inputs achieved the best performance results in all prediction intervals, demonstrating the advantages of using a multidimensional dataset in our model for wind speed and direction prediction. The inclusion of additional meteorological parameters significantly enhanced the predictive accuracy of the model, making it a valuable tool for spatiotemporal meteorological forecasting.

### Model convolutional weight visualization

The CLCRN employs a specialised convolution kernel designed to handle the unique characteristics of meteorological data distributed over a spherical manifold, such as the Earth’s surface. This kernel, known as the CLC kernel, aims to capture the local spatial patterns influenced by the distance and orientation of neighbouring nodes.

The convolution kernels indicate the influence of surrounding neighbor nodes on the central node and approximate meteorological patterns in local regions. Therefore, in this section, we visualize the learnable term $\:MLP\left(\left[{x}_{j}^{{i}^{{\prime\:}}},{x}_{j}\right]\right)$ from Eq. 6 to illustrate how local meteorological patterns propagate across a network of meteorological stations.

**Fig. 4:**
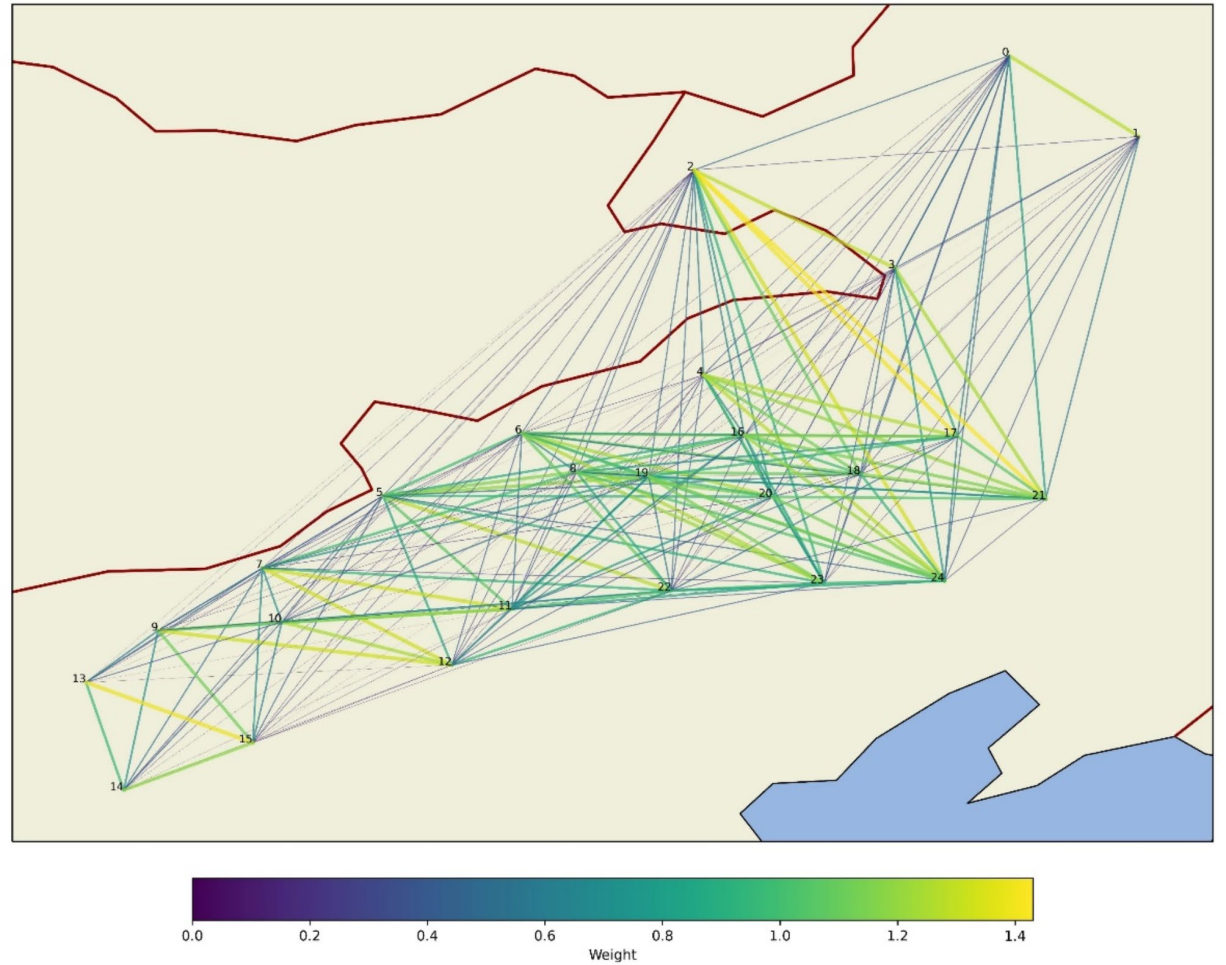
Calculation weights.

The first step in calculating the weights involves constructing a graph where each node represents a meteorological station and the edges represent the connections between these stations. An adjacency matrix is typically constructed using the k-nearest neighbour algorithm based on the induced spherical distance between the nodes. Subsequently, a multi-layer feedforward network was used to approximate these conditional kernel weights. The calculated weights are shown in Fig. 4. In Fig. 4, the colours and thicknesses of the lines connecting the nodes represent the magnitudes of the calculated weights. Heavier and brighter coloured lines indicate stronger influences between nodes, as determined by the kernel function. This visualisation helps understand how local meteorological patterns propagate across a network of meteorological stations.

## Discussion

### Comparison against alternative models

As shown in Tables 2 and 3, this study compared other baseline networks with RNN, such as classical attention-based networks (CLCSTN) by embedding a convolution layer into the framework of the multi-step temporal graph convolutional network (MSTGCN) as an attention-based version of CLCRN. Table 2 presents the MAE and RMSE for various prediction intervals. The models compared included DCRNN, GCoNVGRU, TGCN, STGCN, MSTGCN, CLCSTN, and CLCRN. As shown in Table 2, DCRNN utilises diffusion convolution to capture spatial dependencies and RNNs for temporal dynamics. Although it performs well, its error metrics are generally higher than those of OUR model. Graph convolutional gated recurrent unit (GCoNVGRU) combines graph convolution for spatial data and gated recurrent unit (GRU) for temporal sequences. Its performance is similar to that of the DCRNN but slightly less effective, particularly for longer prediction intervals. Temporal graph convolutional network (TGCN) integrates graph convolution and RNNs to model spatiotemporal data. This model shows higher error metrics compared to DCRNN and GCoNVGRU, indicating less effectiveness in capturing complex spatiotemporal relationships. TGCN integrates graph convolution and RNNs to model spatiotemporal data. This model shows higher error metrics compared to DCRNN and GCoNVGRU, indicating less effectiveness in capturing complex spatiotemporal relationships. MSTGCN is an advanced version of STGCN with multi-step prediction capabilities. Although it performs better than STGCN, its error metrics are still higher compared to other models. CLCSTN uses CLC focused on spatial-temporal dependencies without recurrent units. It shows competitive performance, particularly in short-term predictions, but lags slightly behind CLCRN in longer intervals.

Overall, Graph-based neural networks are commonly used for signals with irregular spatial distributions. In this approach, the nodes on a sphere are represented as nodes in an established graph, enabling fast implementation and good performance^28^. Following the emergence of GNNs, spatiotemporal forecasting models have predominantly relied on graph-based neural networks because of their capacity to learn representations of spatially irregular distributed signals. There are limitations in the design of convolutional kernels within existing graph neural network (GNN) methods^18^. Current graph convolutional approaches for spatiotemporal wind speed prediction typically employ fixed convolutional kernels, which struggle to effectively capture diverse meteorological patterns associated with different geographical locations. The comparison result shows our model consistently achieves lower MAE and RMSE values across all prediction intervals (3, 6, 9, and 12 h), outperforming other models in both short-term and long-term forecasts, demonstrating its robustness and accuracy in predicting wind dynamics.

### Advanced performance via multidimensional inputs

The benchmarking results clearly demonstrate the advantages of a CLC-based model for wind speed and direction prediction. In this section, we delve into a detailed comparison between the CLCRN and the CLCSTN when using multidimensional inputs. We specifically evaluate the differences in performance when utilising five meteorological parameters versus just the sine ‘u’ and cosine ‘v’ components of wind speed. This analysis underscores the impact of incorporating a broader range of meteorological variables into a model’s predictive capabilities.

The original CLCRN model forecasts each selected weather parameter—temperature, humidity, cloud cover, and surface wind components—individually and evaluates their accuracy separately, rather than analysing them as integrated feature inputs. This process ignores the multiple causes of the meteorological characteristics of the wind components. Table 3 focuses on comparing the performance of the CLC-based model using all five meteorological parameters (5D input) against using only the sine ‘u’ and cosine ‘v’ components of wind speed (2D input). This comparison highlights the impact of incorporating additional meteorological variables into the prediction model. For the 5D input results (Tabel 1), the CLC-based model achieves better performance metrics than the 2D input results across all the prediction intervals. The inclusion of air temperature, dew point temperature, and atmospheric pressure provided the model with a richer dataset, enabling it to learn more complex interactions and dependencies between different atmospheric conditions. This comprehensive input allows the CLCRN model to capture subtle variations in wind dynamics that are not evident when only wind-speed components are used.

In contrast to other models such as TGCN, CLC-based models with a 2-D input configuration, which use only the wind speed components, show relatively higher MAE and RMSE values. Although it benefits from the advanced features of the CLC structure, the lack of an additional meteorological context limits its predictive power. The superior performance of the CLC-based model with 5-D inputs can be attributed to several factors. First, the richer data representation from additional meteorological parameters enhances the model’s ability to predict wind dynamics accurately. This enhanced learning capability allows the model to learn complex relationships and interactions between different parameters, leading to more accurate forecasts. Subsequently, the robustness of the model was improved by integrating multiple data sources, making it more resilient to variations and anomalies in the data.

### Interpretability visualization

The weight-based representation in our model offers a detailed perspective on meteorological dynamics, where each variable is analysed within the context of its interactions with others, thereby enhancing our understanding of the complex environmental network that interconnects various regions. Our approach allows for a comprehensive analysis of the interactions between weather stations, thereby enhancing the predictive accuracy of the model by considering not only isolated data points but also the complex web of relationships that exist within the dataset. Therefore, the local convolution kernel of the model is instrumental for both the interpretability and advancement of precision in weather forecasting models. To handle the irregular distribution of nodes, the kernel function includes reweighting terms that adjust the influence of each neighbour based on their relative angles and distances. This ensures that the contributions from neighbours are balanced, even if the spatial distribution is uneven. This mapping preserves the relative distances and orientations, which are crucial for accurately modelling local meteorological patterns.

Observations from the network map in Fig. 4 reveal that the interstation weights are more pronounced along the northwest-southeast axis. This pattern corresponds closely to the dominant wind direction in Inner Mongolia, which is predominantly influenced by seasonal shifts between the Siberian high-pressure system during winter and the East Asian Monsoon during summer. In winter, strong north-westerly winds prevail because of the cold and dry air descending from Siberia. Conversely, in summer, the winds shift predominantly south-easterly, as warmer and moist air from the Pacific influences this region. The heavier weights along the northwest-southeast axis observed in the network visualisation provide a robust validation of the meteorological model’s capability to simulate and predict the actual wind dynamics of the Inner Mongolia region. This correlation between the modelled weights and actual wind patterns underscores the effectiveness of the CLC framework in capturing and representing complex meteorological interactions.

The spatial distribution of the model weight alignment with known wind patterns in Inner Mongolia validates current meteorological models and enhances our understanding of regional weather dynamics. This consistency is indicative of the model’s high fidelity in representing spatial interactions and its utility in practical applications such as renewable energy planning and weather-dependent logistical operations. The evidence provided by the model’s output, demonstrating significant north-westerly influences, paves the way for targeted scientific and industrial applications aimed at harnessing the unique meteorological characteristics of the Inner Mongolian Plateau.

### Performance superiority and limitation

Table 2 presents the effectiveness of our model for spatiotemporal meteorological forecasting. By leveraging CLC and RNNs, our enhanced model outperformed the other models across all prediction intervals. Its ability to capture both spatial and temporal dependencies, coupled with the use of comprehensive meteorological inputs, makes it a superior choice for accurate and robust weather forecasting. Comparing with other models, the advantages of this enhanced model are summarised as follows:

1. Integrated Spatial and Temporal Dynamics: The proposed model combines CLC with RNNs, effectively capturing spatial dependencies through local convolutions and temporal dynamics through recurrent layers. This dual approach enables the model to learn complex patterns in the data, resulting in lower MAE and RMSE values across all prediction intervals.
2. CLC Structure: The CLC mechanism allows our model to adapt its convolutional operations based on local data characteristics. This adaptability enhances the model’s ability to capture unique meteorological patterns in different regions, surpassing the predictive performance of models that rely on standard convolutional approaches.
3. Utilisation of RNNs for Temporal Dependencies: By incorporating RNNs into its architecture, our model can maintain memories of past states and effectively handle temporal dependencies. This capability is particularly beneficial for meteorological forecasting, where understanding the temporal evolution of weather parameters is crucial for accurate predictions. Although CLCSTN also utilises CLC, it lacks the recurrent components present in CLCRN. This omission limits its ability to capture temporal dependencies as effectively as the CLCRN. The integration of RNNs into our model provides an additional layer of temporal modelling capability, which is crucial for accurate long-term forecasting.

This study show the superiority of the proposed Conditional Local Convolution Recurrent Network (CLCRN) model concerning predictive accuracy. However, it is essential to acknowledge certain limitations inherent in our research. Specifically, the model performance is evaluated within the context of specific meteorological conditions characteristic of the northern Inner Mongolia region. While these conditions provide valuable insights into the model’s effectiveness, additional factors, such as extreme weather events—like storms, heavy snowfall, or heatwaves—and localized anomalies, such as microclimates or terrain influences, are not integrated into our analysis. These factors could significantly affect wind dynamics and the accuracy of wind speed predictions.

Meanwhile, Table 3 illustrates CLCRN model exhibits slightly diminished accuracy with 2D inputs relative to the CLCSTN model. This observation indicates that CLCRN model places a greater reliance on multidimensional meteorological data. Consequently, it is evident that integrating multiple parameters is a crucial step in enhancing predictive performance and improving wind speed forecasting accuracy. This observation highlights the need for further research to explore the adaptability of the CLCRN model across diverse meteorological contexts. Future studies should investigate the integration of additional meteorological variables, such as evapotranspiration, pressure altitude, weather system types, or soil moisture, which may further enhance model robustness. Moreover, potential improvements in model architecture—such as the inclusion of advanced feature extraction techniques or hybrid modelling approaches—could lead to significant advancements in predictive accuracy.

## Conclusion

Precise wind speed prediction is an urgent requirement for not only renewable wind resources development, but also physical geographical environmental and ecology protection, especially in large regions on plateau. Recent advances in wind speed prediction have come from combining GNNs with RNNs, addressing dynamic graph challenges in spatiotemporal data. However, these methods often fall short in capturing complex location-specific patterns and integrating multiple meteorological factors. To address these limitations, we propose a model that combines Conditional Local Convolution (CLC) structure for capturing intricate location-specific patterns with a multi-factor embedding layer that integrates multisource data, including temperature, pressure, dew point, and wind components, to predict wind speed, thereby improving prediction accuracy and robustness in diverse meteorological conditions. The results showed that our enhanced CLCRN model consistently achieved lower MAE and RMSE values across all prediction intervals compared to the other models. These modifications resulted in significant improvements in predicting wind speed and direction, demonstrating the effectiveness of integrating additional meteorological variables into the forecasting model. The weight-based representation in the model provides a detailed perspective on meteorological dynamics, enabling a comprehensive analysis of the interactions between weather variables. The consistency of the local convolution weight spatial distribution with known wind patterns in Inner Mongolia provides compelling evidence of the enhanced model’s superior interpretability and utility value for practical applications. This highlights the model’s capability to simulate and predict actual wind dynamics, and offers a promising direction for future applications in various fields requiring precise wind speed predictions.

## Electronic supplementary material

Below is the link to the electronic supplementary material.

Supplementary Material 1

Supplementary Material 2

Supplementary Material 3

## Data Availability

Upon reasonable request, the corresponding author will provide access to the data used to support the study’s findings.
