# Supplementary material for: Spatiotemporal wind speed forecasting using conditional local convolution and multidimensional meteorology features
Source: Sci Rep. 2024 Oct 31;14:26219. doi: 10.1038/s41598-024-78303-8 (PMC11527990; doi:10.1038/s41598-024-78303-8)
Supplement: Supplementary file 1 — Supplementary Material 1 [file 41598_2024_78303_MOESM1_ESM.docx]

## Highlights

- An enhanced Conditional Local Convolution Recurrent Network (CLCRN) model is introduced, improving spatiotemporal wind speed forecasting using multidimensional meteorological inputs.
- This enhanced model consistently achieves lower Mean Absolute Error (MAE) and Root Mean Squared Error (RMSE) values across multiple prediction intervals (3, 6, 9, and 12 hours) compared to other models.
- The spatial distribution of the local convolution weights was consistent with known local wind patterns in Inner Mongolia region, indicating superior model interpretability.
