# Supplementary material for: Spatiotemporal wind speed forecasting using conditional local convolution and multidimensional meteorology features
Source: Sci Rep. 2024 Oct 31;14:26219. doi: 10.1038/s41598-024-78303-8 (PMC11527990; doi:10.1038/s41598-024-78303-8)
Supplement: Supplementary file 2 — Supplementary Material 2 [file 41598_2024_78303_MOESM2_ESM.docx]

List of 4 potential reviewers:

|  | Name | Email | Position and Affiliation | Personal Website |
| --- | --- | --- | --- | --- |
| 1. | Lizhe Wang | lizhe.wang@gmail.com | School of Computer Science, China University of Geosciences (Wuhan) | http://grzy.cug.edu.cn/LizheWang |
| 2. | Hong Tang | hongtang@bnu.edu.cn | Department of Geography Science, Beijing Normal University | https://geot.bnu.edu.cn/Public/htm/news/5/182.html |
| 3. | Fei Yang | yangfei@igsnrr.ac.cn | State Key Laboratory of Resources and Environmental Information System, Institute of Geographic Sciences and Natural Resources Research of Chinese Academy of Sciences | http://www.lreis.ac.cn/kyry/fyjy/201610/t20161010_350242.html |
| 4. | Keming Yang | ykm@cumtb.edu.cn | School of Geoscience and Mapping Engineering, China University of Mining and Technology (Beijing) | https://dcxy.cumtb.edu.cn/info/1010/1652.htm |
