# Supplementary material for: Spatiotemporal wind speed forecasting using conditional local convolution and multidimensional meteorology features
Source: Sci Rep. 2024 Oct 31;14:26219. doi: 10.1038/s41598-024-78303-8 (PMC11527990; doi:10.1038/s41598-024-78303-8)
Supplement: Supplementary file 3 — Supplementary Material 3 [file 41598_2024_78303_MOESM3_ESM.docx]

**COVER LETTER**

Dear Editors:

We are greatly thankful to you and the reviewers for providing insightful comments on our manuscript (Number: 0bada89f-2f39-46ab-9d2a-fb0da0fa127f) entitled "Spatiotemporal Wind Speed Forecasting Using Conditional Local Convolution and Multidimensional Meteorology Features". The comments are valuable and very helpful for the improvement of the quality of our paper. We have revised the manuscript carefully according to the comments and provided a point-to-point response (in blue font) to all comments.

In the latest version of the manuscript, we have made significant improvements based on the reviewers' constructive feedback. We have revised the abstract to offer a clearer summary of our study, emphasizing our innovative contributions, including the integration of multidimensional meteorological data and the development of the enhanced Conditional Local Convolutional Recurrent Network (CLCRN) model. We expanded the methodology section to provide detailed descriptions of the CLCRN model architecture, including the multi-factor embedding layer and the specific parameter settings for the Conditional Local Convolution (CLC). Additionally, we clarified the incorporation of wind direction in our analysis by decomposing wind speed records into their directional components. We also ensured that all model abbreviations in the tables were defined for clarity. The preprocessing section was revised to detail how we utilized a sliding window approach with a specified window size and stride for constructing our dataset, ensuring all meteorological features accurately reflect temporal dynamics. Lastly, we provided a comprehensive comparison of model parameters and structures in experimental results section.

We hope you will now view our paper favorably for publication in *Scientific Reports*. If you have any queries, please don’t hesitate to contact us.

Best regards,

Yours sincerely,

Juanle Wang ([wangjl@igsnrr.ac.cn](mailto:wangjl@igsnrr.ac.cn))

2024.10.14
